# Identification of bromelain subfamily proteases encoded in the pineapple genome

**DOI:** 10.1038/s41598-023-38907-y

**Published:** 2023-07-18

**Authors:** Ashley G. Yow, Hamed Bostan, Roberto Young, Giuseppe Valacchi, Nicholas Gillitt, Penelope Perkins-Veazie, Qiu-Yun (Jenny) Xiang, Massimo Iorizzo

**Affiliations:** 1 Department of Horticultural Science, North Carolina State University, Raleigh, NC 27695 USA; 2 Plants for Human Health Institute, North Carolina State University, Kannapolis, NC 28081 USA; 3 Research Department of Dole, Standard Fruit de Honduras, Zona Mazapan, La Ceiba, 31101 Honduras; 4 Department of Environmental Sciences and Prevention, University of Ferrara, Ferrara, Italy; 5 Berkley LLC, Kannapolis, NC 28081 USA; 6 Department of Plant and Microbial Biology, North Carolina State University, Raleigh, NC 27695 USA

**Keywords:** Agricultural genetics, Functional genomics, Genomics, Plant genetics, Plant sciences, Plant genetics, Plant molecular biology

## Abstract

Papain (aka C1A) family proteases, including bromelain enzymes, are widespread across the plant kingdom and play critical regulatory functions in protein turnover during development. The proteolytic activity exhibited by papain family proteases has led to their increased usage for a wide range of cosmetic, therapeutic, and medicinal purposes. Bromelain enzymes, or bromelains in short, are members of the papain family that are specific to the bromeliad plant family. The only major commercial extraction source of bromelain is pineapple. The importance of C1A family and bromelain subfamily proteases in pineapple development and their increasing economic importance led several researchers to utilize available genomic resources to identify protease-encoding genes in the pineapple genome. To date, studies are lacking in screening bromelain genes for targeted use in applied science studies. In addition, the bromelain genes coding for the enzymes present in commercially available bromelain products have not been identified and their evolutionary origin has remained unclear. Here, using the newly developed MD2 v2 pineapple genome, we aimed to identify bromelain-encoding genes and elucidate their evolutionary origin. Orthologous and phylogenetic analyses of all papain-family proteases encoded in the pineapple genome revealed a single orthogroup (189) and phylogenetic clade (XIII) containing the bromelain subfamily. Duplication mode and synteny analyses provided insight into the origin and expansion of the bromelain subfamily in pineapple. Proteomic analysis identified four bromelain enzymes present in two commercially available bromelain products derived from pineapple stem, corresponding to products of four putative bromelain genes. Gene expression analysis using publicly available transcriptome data showed that 31 papain-family genes identified in this study were up-regulated in specific tissues, including stem, fruit, and floral tissues. Some of these genes had higher expression in earlier developmental stages of different tissues. Similar expression patterns were identified by RT-qPCR analysis with leaf, stem, and fruit. Our results provide a strong foundation for future applicable studies on bromelain, such as transgenic approaches to increase bromelain content in pineapple, development of bromelain-producing bioreactors, and studies that aim to determine the medicinal and/or therapeutic viability of individual bromelain enzymes.

## Introduction

With over 27 tons of pineapples produced globally in 2020, amounting to nearly $9 billion US in value (https://www.fao.org/faostat/), pineapple is one of the most economically and culturally important tropical fruits worldwide. Demand for pineapple has increased annually since the 1990s and is projected to continue increasing^1, 2^. Properties of pineapple driving demand include its nutritional importance as a dietary significant source of several key nutrients including vitamin C, manganese, fiber and potassium, as well as being the only commercial source of bromelain, a proteolytic enzyme with high therapeutic value^2–4^. Some examples of therapeutic uses of bromelain include relieving digestive inflammation, decreasing post-surgical inflammation, and wound debridement^5–7^.

Bromelain enzymes break peptide bonds with a cysteine active site, and are therefore classified as cysteine proteases^8–11^. Plant cysteine proteases are a large family of proteins that perform diverse cellular functions during development. Many of the therapeutically and economically important cysteine proteases, including pineapple bromelains, are classified as members of the protease subfamily C1, clan CA, and are therefore referred to as C1A proteases (also known as papain-family proteases)^9, 11, 12^. C1A proteases are a broad class of proteolytic enzymes that function to prevent fungal and bacterial disease, deter herbivory, and degrade proteins in order to maintain proper plant growth and development^12–17^. These proteases, in combination with their inhibitors, accumulate throughout various plant tissues and are often found in high abundance in ripening fruit^17^, suggesting they play an important role in fruit maturation. In addition, pineapple stem and leaf tissue serves as the main source of commercial bromelain extraction^6^ because bromelain accumulates at higher levels in the stem than in the fruit and it allows pineapple growers to utilize plant “waste” material after the fruit is harvested.

Previous studies have greatly advanced knowledge about the pineapple bromelain gene family. Some of these efforts include determining the number of C1A protease in the pineapple genome^18, 19^, characterizing the structural properties and conformation of C1A protease proteins^20–25^, and further sub-classifying these proteins into subgroups including putative bromelain proteins^18^. It has been reported that slight differences in amino acid sequence contribute to unique substrate and inhibitor-binding properties among C1A family proteases in pineapple^26^, which may, in turn, reflect neo- or sub-functionalization of bromelains. Two studies have reported 61 or 62 C1A family proteases in the pineapple genome^18, 19^, and one of these studies classified them into 9 subfamilies based on phylogeny with those in other species. Expression characterization of previously identified C1A protease genes and enzymatic assays using multiple distinct tissue types has strongly suggested that this protease family is heavily involved in fruit ripening^18, 19, 27^. Researchers have sequenced a stem bromelain protein and an ananain protein from pineapple stem extracts and evaluated their structural and enzymatic properties^26, 28^. Up to eight distinct catalytically active cysteine proteases have been identified in a commercial stem bromelain powder^21^. Despite these advancements, it is still uncertain which sub-group(s) of genes specifically encode the bromelain enzymes within the C1A protease family in pineapple and which genes code for bromelain proteins used in therapeutic studies and/or present in bromelain products. This lack of knowledge limits selection of genes to study bromelain function, trace their ancestry, or develop a strategy to increase protease accumulation and/or therapeutic properties.

To fill this gap and complement previous research, a combination of genomic, transcriptomic, and proteomic data was used in this study for sub-classification of C1A protease genes in pineapple and to create a link between genes in the pineapple genome and bromelain enzymes in commercial products and to trace their ancestry. Data from this study could be used for future applied research that requires strong foundational data such as gene cloning, transgenic approaches to increase or induce bromelain production in plant bioreactors, genomic-assisted breeding, or for targeted studies that evaluate therapeutic effects of specific bromelains.

## Results

### C1A protease family genes in the pineapple MD2 v2 genome

**Figure 1:**
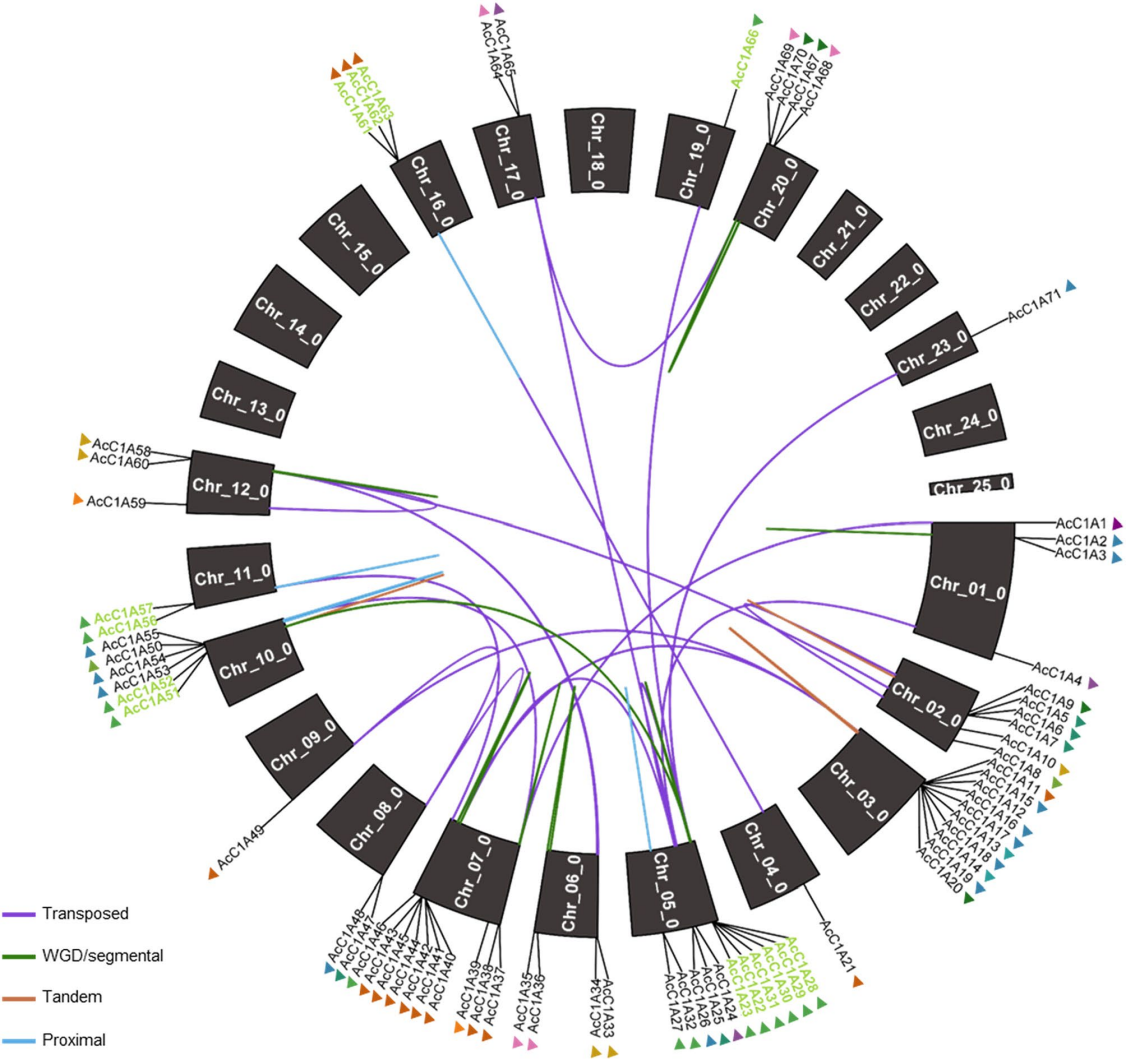
Circular plot illustrating the location and duplication mechanism of all papain (C1A) family protease genes identified in the MD2 v2 genome. Line colors correspond to duplication type, names in green indicate members of orthogroup 189, and triangle colors correspond to phylogenetic clade (see Fig. 2).

Presence of either the C1 peptidase or I29 inhibitor domains were used as a signature to identify genes belonging to the C1A protease gene family^9^. 71 C1A genes were identified (*AcC1A1*–*AcC1A71*), and were distributed across 17 pineapple chromosomes (Fig. 1, Supplementary Table S1).

**Figure 2:**
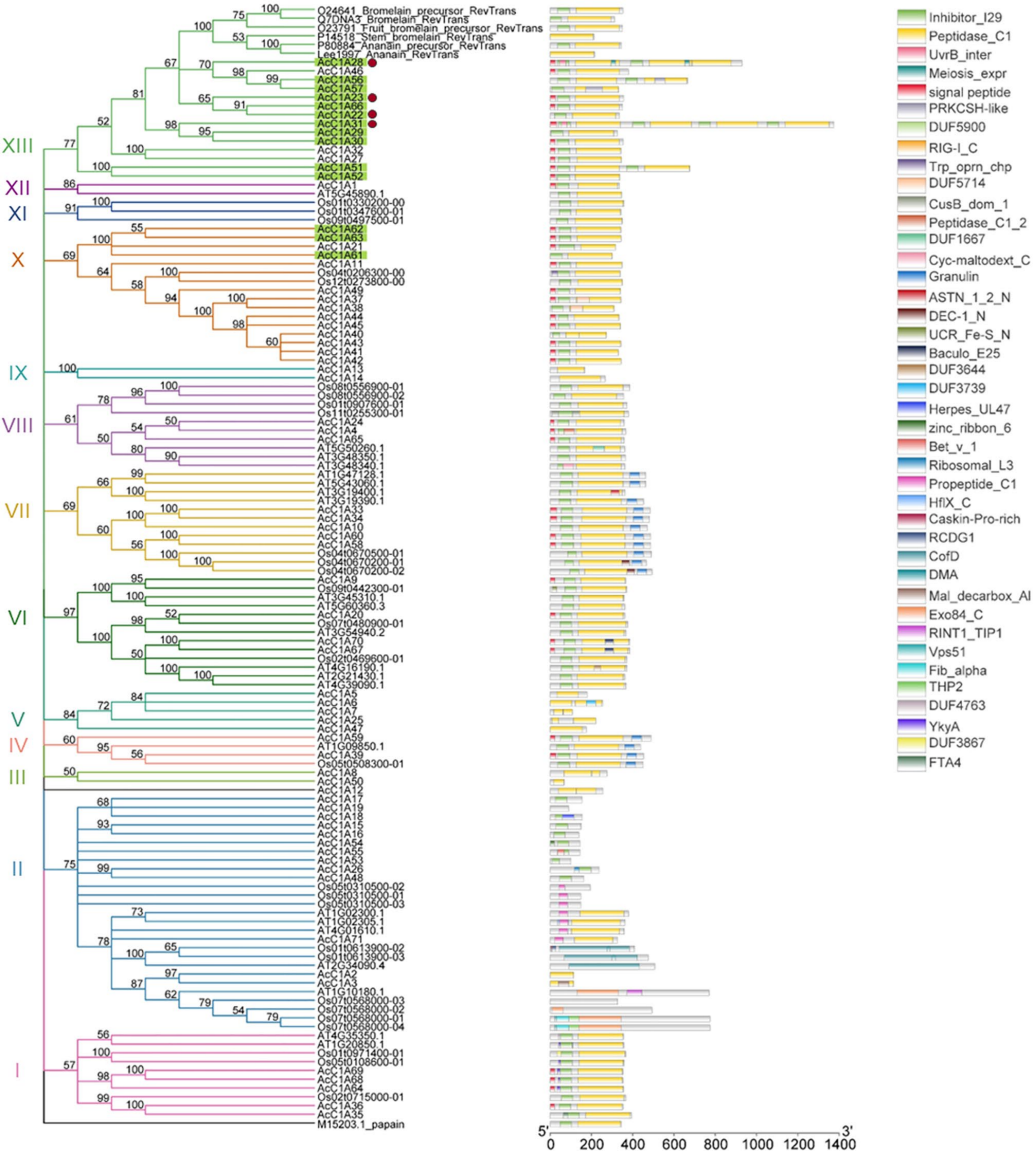
Phylogenetic tree depicting the relationship between C1A protease genes identified in pineapple MD2 v2, *Arabidopsis*, and rice, as well as previously published bromelain sequences. Branches are colored according to phylogenetic clade. Green highlight indicates C1As placed into orthogroup 189. Dark red circles indicate those found in commercial bromelain products by proteomic analysis.

PacBio iso-seq and Illumina data were used to verify the structure of the predicted MD2 v2 C1A genes (see Supplementary Fig. S1). For instance, the gene *ACMD2v2_02.26390* (*AcC1A9*) was predicted as two genes in the F153 v3 genome annotation, however Illumina and PacBio Iso-Seq data supported the prediction of a single gene model (see Supplementary Fig. S1). Manual curation resulted in the improvement of the gene structure of five C1A genes (*ACMD2v2_05.00101 (AcC1A22)*, *ACMD2v2_05.00102* (*AcC1A23*), *ACMD2v2_07.18635* (*AcC1A38*), *ACMD2v2_10.11764* (*AcC1A55*), and *ACMD2v2_11.08385* (*AcC1A57*)), including one that gained a full-length inhibitor I29 domain and four that gained either the C1 peptidase domain or the inhibitor I29 domain. After manual curation, 61 genes contained the C1 peptidase domain, 58 contained the I29 domain, and 53 contained both domains (Supplementary Table S2). Compared to the most recent study on C1A genes in pineapple, which identified 61 total C1As in cultivar ‘F153’, ten new C1A genes were identified in this study^18^. No isoforms were found for the MD2 v2 C1A genes.

### Evolutionary relationships of C1A protease genes in pineapple

The evolutionary relationships of C1A protein sequences were explored through orthologous and phylogenetic analysis. Known bromelain family protein sequences from Uniprot^28^ (Table 1) and an additional published study^26^ were included in these analyses to aid in the classification of pineapple C1As. Orthologous analysis among 9 species representing diverse plant families with high-quality genome annotations resulted in 34,402 total orthogroups. Thirty-seven orthogroups contained the 71 C1A proteases from the pineapple MD2 v2 genome annotation (Supplementary Table S3). Three orthogroups contained C1As from only monocots and twenty-three were specific to pineapple. Among orthogroups containing C1A proteases from pineapple and other species, the number of pineapple C1As was expanded. Within the 37 C1A-containing orthogroups, there were 22 proteins from *Arabidopsis* and 20 from rice. Orthogroup 189 included the published bromelain proteins as well as 15 proteins from MD2 v2 (Supplementary Tables S3 and S4). Out of the 71 predicted C1A proteins in MD2 v2, 17 and 18 had rice and *Arabidopsis* orthologs, respectively, and thirteen were orthologs with both species (Supplementary Table S5). Eighteen pineapple C1As were likely among the early members of the C1A protease family in pineapple as determined by their orthology with proteins in a distantly related dicot species such as *Arabidopsis*.

**Table 1:** Summary of Uniprot protein sequences used in this study.

Uniprot ID	Description	Contains I29 domain?	Predicted subcellular location(s)	Orthogroup	Present in commercial product?^a^	Citation
O23791	Fruit bromelain precursor	Yes	Secreted (highly likely)	189	Yes	Muta et al. (1993)^b^
O24641	Bromelain precursor	Yes	Secreted (highly likely)	189	No	Muta, E., Okamoto, Y. and Ota, S. (1994)^b^
P14518	Stem bromelain	No	no prediction	189	Yes	Ritonja et al. (1989)
Q7DNA3	Bromelain	Yes	Nucleus	189	No	Muta et al. (1993)^b^
P80884	Ananain precursor	Yes	no prediction	189	Yes	Robertson, C. E. and Goodenough, P.W. (1997)^b^

^a^Determined by proteomic analysis.

^b^Direct submission of protein sequence to Uniprot database.

Three of the pineapple C1As in orthogroup 189 (AcC1A61, AcC1A62, AcC1A63) were orthologous to one *Arabidopsis* (AT2G34080.1) and one rice (Os01t0613500-01) protein sequence. However, none of the other pineapple C1As in orthogroup 189 had orthologs with any of the species included in the orthologous analysis. One of the pineapple proteins (ACMD2v2_16.26903) in orthogroup 189 was not functionally characterized as a C1A protease in this study because it lacked the C1 peptidase and I29 inhibitor domains that are characteristic of the C1A protease family, but it shared sequence homology with a senescence-specific cysteine protease. It is possible that this gene shared ancestry with pineapple C1A genes, however, it may have not accumulated the necessary mutations to gain the conserved C1 peptidase and I29 inhibitor domains in the corresponding protein.

We examined the congruence between orthologous clustering results presented here and the previously published bromelain subfamilies (referred here as “F153 C1As”)^18^. Overall, pineapple ‘F153’ C1As that were within the same orthogroup in this study were also previously clustered into the same bromelain subfamily (Supplementary Table S6). Previous attempts to identify putative bromelain genes utilized only phylogenetic analysis, which clustered pineapple C1A proteases into 9 distinct phylogenetic clades, but were unable to distinguish bromelain subfamily from papain family genes^18^. Therefore, this study improves upon previous work by utilizing a combination of data, including orthology, phylogeny, and proteomic analysis for identifying bromelain subfamily protease genes. Additionally, 43 of the 62 C1A family proteases that were previously identified in the pineapple ‘F153’ genome^19^ were orthologous to C1A proteases identified here in ‘MD2’. The results presented here complement previous findings that demonstrate how large gene families diverge into subfamilies.

Phylogenetic analysis sorted pineapple C1A proteases into 13 clades (I–XIII) (Fig. 2). Across all C1As identified, proteins within the same orthogroup were largely placed into the same phylogenetic clade (Supplementary Table S5). However, C1A proteins from orthogroup 189 were grouped in two clades (X and XIII), indicating that members of this orthogroup have diverged. Besides orthogroup 189 C1As from MD2 v2, clade XIII also included all previously functionally characterized fruit bromelain, stem bromelain, and ananain sequences. MD2 v2 C1A sequences that clustered very closely with known bromelains included AcC1A28, AcC1A46, AcC1A56, AcC1A57, AcC1A23, AcC1A66, and AcC1A22, therefore, particular focus was placed on these proteins and their encoding genes in subsequent analyses. It is also worth noting that phylogenetic clade XIII only contained protein sequences from pineapple, further indicating that clade XIII represents proteins belonging to the bromelain subfamily, which is specific to the Bromeliaceae plant family. Overall, the results of the orthologous and phylogenetic analysis suggested that pineapple bromelain proteins belonging to orthogroup 189, clade XIII diverged from other C1A proteases and are the most likely bromelain subfamily proteases.

### C1A protein content of pineapple stem

To verify the presence and relative abundance of identified putative bromelain proteins in pineapple stem, the major extraction source of commercial bromelain enzymes, a proteomic analysis was first performed on two commercially available bromelain products (B1 and B2). A total of 21 putative bromelain proteins were identified by peptide sequence tags (see “Methods”) across the two bromelain samples analyzed. However, 8 of them had matches to < 3 peptide tags, leaving 13 proteins that were considered high-confidence peptides (FDR < 5% and ≥ 3 peptide sequence tags) and could reliably be considered as present in the samples analyzed (Table 2). Out of these 13 proteins that were identified by peptide sequence tags in the B1 and B2 samples, 3 corresponded to known bromelains obtained from Uniprot (O23791 (fruit bromelain precursor), P14518 (stem bromelain), and P80884 (ananain precursor)) and 4 corresponded to putative bromelains predicted in the MD2 v2 genome (AcC1A22, AcC1A23, AcC1A28, AcC1A31). The remaining 6 proteins identified by peptide sequence tags in the B1 and B2 samples correspond to actin (ACMD2v2_02.26173), a GOS9-like protein (ACMD2v2_13.22649), a polygalacturonase inhibitor 1 precursor (ACMD2v2_03.20028), and C1A protease family inhibitors (ACMD2v2_03.18543, ACMD2v2_13.22774, ACMD2v2_17.22170). All proteins identified in the B1 and B2 samples were annotated using the top BLAST hit.

**Table 2:** Results for proteomic analysis performed on two commercially available stem bromelain samples.

Protein ID	Coverage (%)	Number of peptides	Number of Unique Peptides	Number amino acids	MW (kDa)	B1 (Sigma-Aldrich) %	B2 (Galeno Srl) %	Top BLAST hit
AcC1A31	17	32	32	1375	152.7	21.44	11.98	Fruit bromelain
AcC1A23	51	30	11	356	39.5	21.61	60.43	FBSB precursor
P14518*	70	26	10	212	22.8	0.42	11.93	–
P80884*	50	24	22	345	38.2	7.20	14.12	–
ACMD2v2_03.20028	31	8	8	284	30.5	Not found	0.07	Polygalacturonase inhibitor 1 precursor
ACMD2v2_03.18543	25	5	5	272	30	22.58	0.49	Bromelain inhibitor
AcC1A28	7	4	4	929	102.5	1.63	0.01	Fruit bromelain
ACMD2v2_13.22649	41	4	4	132	14	3.84	Not Found	Protein GOS9-like
ACMD2v2_17.22170	33	4	4	132	14.7	Not found	0.05	Cysteine proteinase inhibitor 10
O23791*	13	4	4	351	39	19.70	0.14	–
ACMD2v2_02.26173	10	3	3	377	41.6	0.19	0.01	Actin
AcC1A22	15	3	2	230	24.8	0.06	0.01	Fruit bromelain
ACMD2v2_13.22774	17	3	3	187	21.1	Not found	0.01	Pineapple cystatin

The table includes proteins identified as present in one or both samples and their relative abundances. Protein sequences used for development of peptide sequence tags included published sequences from the Uniprot protein database (*) and predicted protein sequences from the pineapple MD2 v2 genome. Results were filtered to show only proteins identified by ≥ 3 peptide sequence tags.

Assessment of the relative abundance of the proteins detected in the B1 and B2 samples was done by comparing mass spectrometry data from the two samples. These results showed that in the B1 sample, a bromelain inhibitor (ACMD2v2_03.18543) accounted for 22.58% of the total protein content, followed by C1A proteases AcC1A23 (21.61%), AcC1A31 (21.44%), AcC1A28 (1.63%), and AcC1A22 (0.06%). In addition, the GOS9-like protein (ACMD2v2_13.22649) and actin (ACMD2v2_02.26173) were present in the sample at 3.84% and 0.19%, respectively. Uniprot sequences (O23791, P14518, and P80884) were also identified in the B1 sample, ranging from 19.7% for O23791 to 0.42% for P14518. In the B2 sample, C1A proteases AcC1A23 accounted for 60.43% and AcC1A31 accounted for 11.98%, while AcC1A28 and AcC1A22 each accounted for only 0.01% of total protein content; bromelain inhibitor accounted for 0.49% and actin accounted for 0.01%. The GOS9-like protein was not present in the B2 sample, however, three other proteins were identified that were not present in sample B1, including a polygalacturonase inhibitor 1 precursor (ACMD2v2_03.20028) at 0.07%, cysteine proteinase inhibitor 10 (ACMD2v2_17.22170) at 0.05%, and three-dimensional structure of pineapple cystatin (ACMD2v2_13.22774) at 0.01% of total protein content. Although 5 proteins were identified in the B2 sample that were not C1A proteases, the sum of their relative abundance still only amounted to 0.63% of the total protein content, whereas the relative abundance of non-C1A proteins in the B1 sample summed to 26.61%. Interestingly, the majority of the difference was accounted for by the large amount of bromelain inhibitor. Along with putative bromelains, cysteine protease and/or bromelain specific inhibitors were also present in both samples analyzed. The detection of C1A protease inhibitors was expected since bromelain inhibitors are known to co-occur with their targets in crude pineapple extracts^12, 29–33^. These observations confirm the robustness of the proteomic analysis.

All of the MD2 v2 C1A proteases identified in the B1 and B2 samples through proteomic analysis were in phylogenetic clade XIII and orthogroup 189, and they are relatively abundant. This result demonstrated that putative bromelains identified through orthologous and phylogenetic analyses were present in a commercial bromelain product and confirmed that AcC1A22, AcC1A23, AcC1A28, and AcC1A31 are likely true bromelains.

### Mode of duplication and ancestry of bromelain genes

The genesis of new genes or gene functions through duplication plays a major role in plant adaptation and phenotypic diversification^34^. To understand the origin of putative bromelain genes, their mode of duplication and the synteny that this gene family shares with a distantly related dicot (*Arabidopsis*) and a more closely related monocot species (rice) was studied. Mode of duplication results revealed that the C1A protease family in pineapple expanded primarily through the transposed duplication mechanism, followed by WGD or segmental, tandem, and proximal mechanisms (Supplementary Table S7).

**Figure 3:**
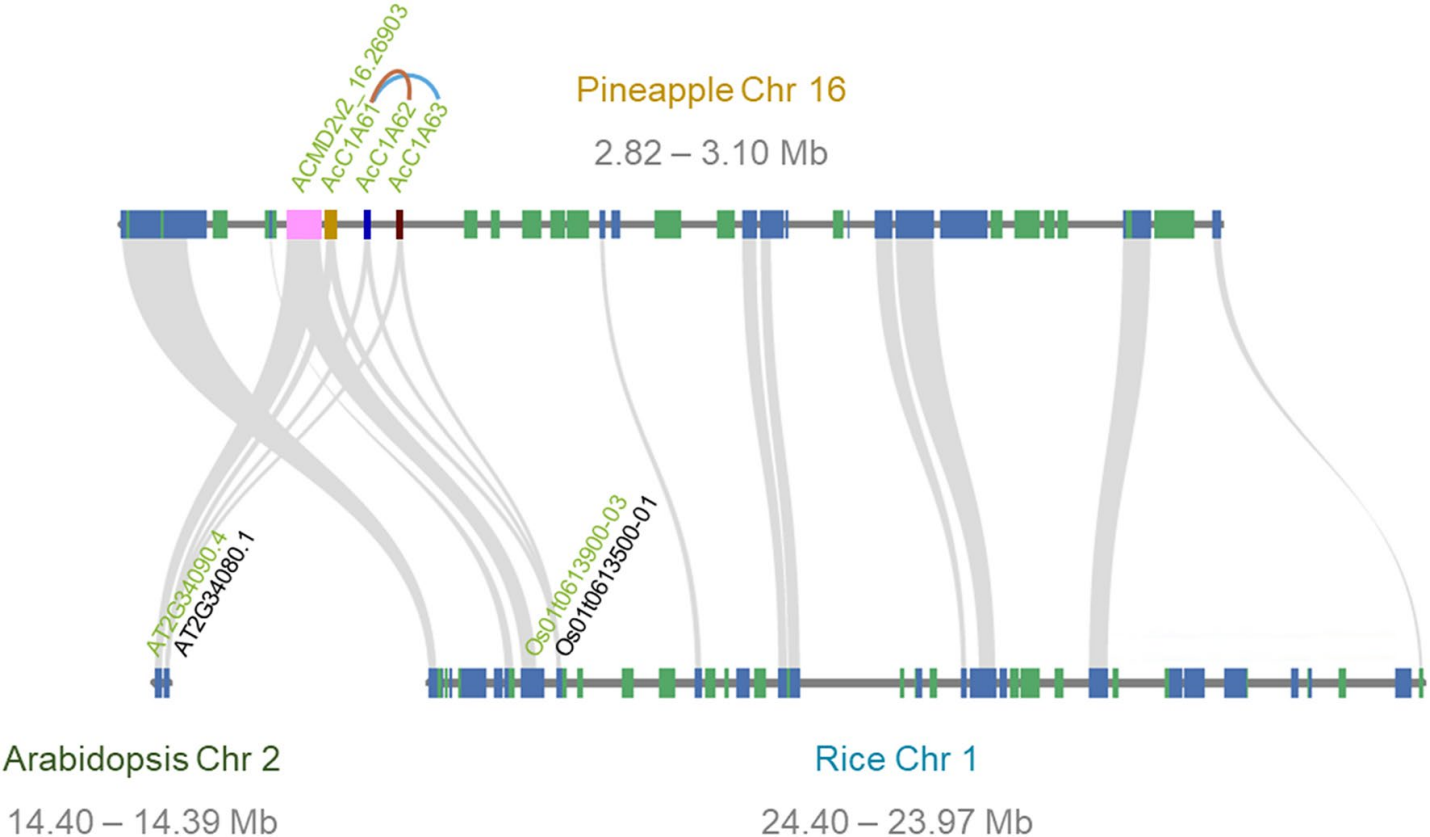
Microsynteny plot showing syntenic genes between pineapple (MD2 v2), *Arabidopsis* (Araport 11), and rice (IRGSP-1.0 2021-11-11) genomic regions. A total of four pineapple C1A protease genes were within the selected syntenic block. One pineapple gene (ACMD2v2_16.26903) was syntenic with one gene from each *Arabidopsis* and rice. The other three pineapple genes (AcC1A61, AcC1A62, and AcC1A63) were all syntenic with one gene from each *Arabidopsis* and rice, serving as an example of how this gene family expanded in pineapple. Genes with green names belong to orthogroup 189.

Through synteny analysis we examined the origin of the genes in orthogroup 189. Synteny analysis between pineapple and *Arabidopsis* genomes identified 10 syntenic blocks containing 15 C1A protease genes and synteny analysis between pineapple and rice genomes identified 14 syntenic blocks containing 18 C1A protease genes. Four pineapple MD2 v2 genes in orthogroup 189 (*ACMD2v2_16.26903, AcC1A61, AcC1A62, AcC1A63*) were syntenic with genes in both *Arabidopsis* and rice (Fig. 3). These genes in pineapple that are syntenic with genes in *Arabidopsis* and/or rice may represent early members of the C1A protease gene family in pineapple. The remaining pineapple MD2 v2 genes in orthogroup 189 may represent C1A proteases subsequently derived in the pineapple family lineage.

To determine if duplicate gene pairs within the C1A protease family in pineapple were undergoing positive, purifying, or no selection, *Ka/Ks* ratios of all gene pairs were calculated. *Ka/Ks* ratios of > 1 indicate genes that have undergone positive selection and are more likely to have potentially gained new functions as a result of higher rates of non-synonymous mutations that alter protein structure. The *Ka/Ks* ratios across all gene pairs identified among the MD2 v2 C1A protease genes indicate that 28 have undergone purifying selection (*Ka/Ks* < 1), while 14 have undergone positive selection (*Ka/Ks* > 1) (Supplementary Table S7). Four of the MD2 v2 C1A genes had *Ka/Ks* = 1, meaning no selection occurred for those genes.

Differences in *Ka/Ks* ratios were observed between types of duplication mechanisms, with transposed duplication having the highest average ratio (1.29), followed by tandem duplication (1.08), proximal (0.78), and WGD or segmental duplication having the lowest average ratio (0.43), indicating that the transposed duplication mechanism may have played a large role in the diversification of the C1A protease gene family in pineapple. Indeed, these results are reflected in the phylogenetic tree; compared to other duplication modes, genes that arose via transposed duplication were more likely to be in a different phylogenetic clade than their progenitors (Fig. 1, Supplementary Table S7). For example, out of 21 total genes that arose via transposed duplication, ten diverged enough to be placed into a different phylogenetic clade than their progenitor genes while eleven clustered into the same clade as their progenitor genes. However, genes that arose via all other duplication mechanisms remained in the same phylogenetic clade as their progenitor gene. Transposed duplication contributed to the expansion of ten phylogenetic clades (I, II, III, IV, V, VII, VIII, X, XII, XIII), which range in size from only two (clade III) to fourteen (clades X and XIII) MD2 v2 genes.

Orthogroup 189, that was associated with the known bromelain proteins, contained 15 C1As from pineapple MD2 v2, but contained only 1 gene from all other species analyzed (Tables S3 and S4). The expansion of this orthogroup likely contributed to the diversity of C1A genes in pineapple. Therefore, we examined the members of this group more closely. Interestingly, genes in orthogroup 189 derived primarily from tandem duplication, followed by transposed and WGD or segmental, then proximal duplications. Some genes, such as *AcC1A28*, were identified as the progenitor for multiple separate duplication events, giving rise to multiple duplicate genes (Fig. 1, Supplementary Table S7).

Among the ten duplicate gene pairs in orthogroup 189, five had *Ka/Ks* < 1, four had *Ka/Ks* > 1 and one had *Ka/Ks* = 1. The average *Ka/Ks* ratio for gene pairs in orthogroup 189 was 1.33, indicating that overall, genes in this orthogroup went through positive selection. It is also worth noting that the putative and likely bromelain genes identified in this study (*AcC1A22, AcC1A23, AcC1A28, AcC1A31, AcC1A66*) had *Ka/Ks* ratios ranging from 1 to 5.33, therefore, they likely underwent diversifying selection to gain new protein functions. Previous studies on gene duplication mechanisms in plants have shown that transposed duplication results in greater divergence in gene expression, amino acid sequence, and promoter region sequence when compared to other modes of gene duplication^35–38^.

Based on duplication mechanism analysis, gene *AcC1A61* was the progenitor of genes *AcC1A62* and *AcC1A63*. The close relationship between these three pineapple genes explains why they all share synteny with only a single *Arabidopsis* (*AT2G34080.1*) and a single rice (*Os01t0613500-01*) gene, and also provides an example for how orthogroup 189 expanded in pineapple. The combination of orthology, duplication mechanism, and synteny analysis results suggested that *AcC1A61* may be the most ancestral member of orthogroup 189. Genes *AcC1A61*, *AcC1A62*, and *AcC1A63* were also placed into a different phylogenetic clade (clade X) than other members of orthogroup 189 (clade XIII) (Fig. 2).

### C1A protease gene expression patterns

Available RNA-seq data were used to evaluate tissue-specific expression, expression level, and differential expression of MD2 v2 C1A genes. Particular attention was paid to gene expression patterns of C1A proteases in orthogroup 189 and the similarities and differences in expression patterns between duplicate gene pairs. This analysis revealed that 27 C1A genes were expressed in all tissues (see “Methods”) and 13 were not expressed (Supplementary Table S8). The remaining 31 C1A genes were expressed in a tissue-specific manner, primarily in one or more of the following tissues: fruit, leaf, root, stem, and/or anther tissues. Reproductive tissues also had notably higher expression levels of the C1A genes that were expressed in all tissues.

Differential expression analysis across 11 different pineapple tissues (bract, core, flower disk, leaf, ovary wall, ovule, placenta, receptacle, root, sepal, and stem) from cv. Shen Wan collected during fruit development^41^ revealed that 25 total C1A genes were differentially expressed, 6 of which were up-regulated in fruit core tissue and 10 were up-regulated in stem tissue. Differential expression analysis across 4 different tissues (flower, one-month-old and two-month-old ripening fruit, and leaf) in *A. bracteatus* ‘CB5’ revealed 17 differentially expressed C1A genes (C1A DEGs). Of these, 8 were up-regulated in one-month-old fruit tissue and 7 were up-regulated in two-month-old fruit tissue. In total, 31 C1A DEGs were identified across both RNA-seq datasets and 11 of those were common to both datasets. Overall, up-regulated genes had higher average expression levels in reproductive (i.e., flower and fruit) tissues than in stem tissue. A higher average expression level was also observed in fruit flesh compared to fruit core tissue, indicating that fresh pineapple fruit represents a good source of bromelain enzymes. Leaf and root tissues consistently had the lowest levels of C1A gene expression across genotypic backgrounds. These results suggest that bromelain enzymes may play an important role in pineapple reproduction and fruit development.

Six of the C1A DEGs that were up-regulated in fruit or stem tissue (*AcC1A22*, *AcC1A23*, *AcC1A28*, *AcC1A30*, *AcC1A31*, *AcC1A66*) were common between both RNA-seq datasets. Interestingly, all of these genes were putative bromelains identified here based on orthologous, phylogenetic, and proteomic analyses. While they were expressed in all tissues, they were expressed significantly higher in core and stem tissues compared to leaf and root tissues and were also highly expressed in different reproductive tissues. The six genes shared similar FPKM expression patterns, however, minor differences could be observed. Additionally, gene expression levels were higher in earlier stages of fruit development than in later stages, but peak expression levels were observed in middle stages, suggesting that these genes play an important role in fruit ripening. The results presented here agree with previous studies that confirmed that bromelain plays a major role in fruit ripening^17, 18^. Gene duplication analysis determined that the likely bromelain *AcC1A28* served as a progenitor gene that gave rise to two other likely bromelains (*AcC1A22*, *AcC1A23*) and a putative bromelain (*AcC1A66*) during duplication events (Supplementary Table S7). Gene expression analysis demonstrated that putative bromelains (i.e., bromelain proteins grouped into orthogroup 189 and phylogenetic clade XIII, but not detectable in bromelain products using proteomic analysis) and likely bromelains (i.e. bromelain proteins grouped into orthogroup 189 and phylogenetic clade XIII and are also found in bromelain products) share strong similarities in expression at the mRNA level. Likely bromelain genes *AcC1A22* and *AcC1A23* shared the most similarities in expression pattern, followed by *AcC1A28* and *AcC1A66* also sharing high similarity in expression pattern with these 2 genes. The putative bromelain gene *AcC1A30* and likely bromelain gene *AcC1A31* had higher similarity in expression pattern with each other than with other C1A DEGs, however these 2 genes also shared high similarity in expression pattern with putative bromelain gene *AcC1A29*. Gene *AcC1A29* gave rise to gene *AcC1A30*, which then gave rise to gene *AcC1A31* during two separate tandem duplication events in pineapple. When examining the relationship between expression patterns and ancestry of the C1A DEGs, results suggested that duplicate genes tended to maintain a similar expression profile as their progenitor gene (Supplementary Table S8), however, minor changes in gene expression may have occurred over time as a result of sub-functionalization of genes in the same family. It is also worth noting that protein sequences for all 6 of the common C1A DEGs and gene *AcC1A29* were placed into orthogroup 189 and the phylogenetic clade containing known bromelain sequences (clade XIII), confirming the functional importance of that orthogroup and phylogenetic clade.

Corresponding proteins of 4 of the C1A DEGs (*AcC1A22*, *AcC1A23*, *AcC1A28*, *AcC1A31*) were also found to be present in stem extract samples through proteomic analysis (Table 2). The combined results of the proteomic and gene expression analyses suggested that these 4 pineapple C1As were expressed at the mRNA and protein levels, providing further validation that these are likely bromelain genes.

### RT-qPCR validation

Relative expression levels for a subset of putative and likely bromelain-encoding genes (*AcC1A22*, *AcC1A23*, *AcC1A66*, *AcC1A51*) (Supplementary Table S9) was determined by RT-qPCR in leaf, stem, and fruit tissue harvested at 6 developmental stages (7 for fruit). Two of these genes (*AcC1A22*, *AcC1A23*) encoded likely true bromelains, one (*AcC1A66*) encoded a putative bromelain, and one (*AcC1A51*) encoded a C1A protease with consistently low levels of gene expression across all tissues as measured by FPKM (Supplementary Table S8). Statistical analysis of the normalized expression values revealed significant differences in gene expression between developmental stages for *AcC1A22*, *AcC1A23*, and *AcC1A66* (Supplementary Table S10).

**Figure 4:**
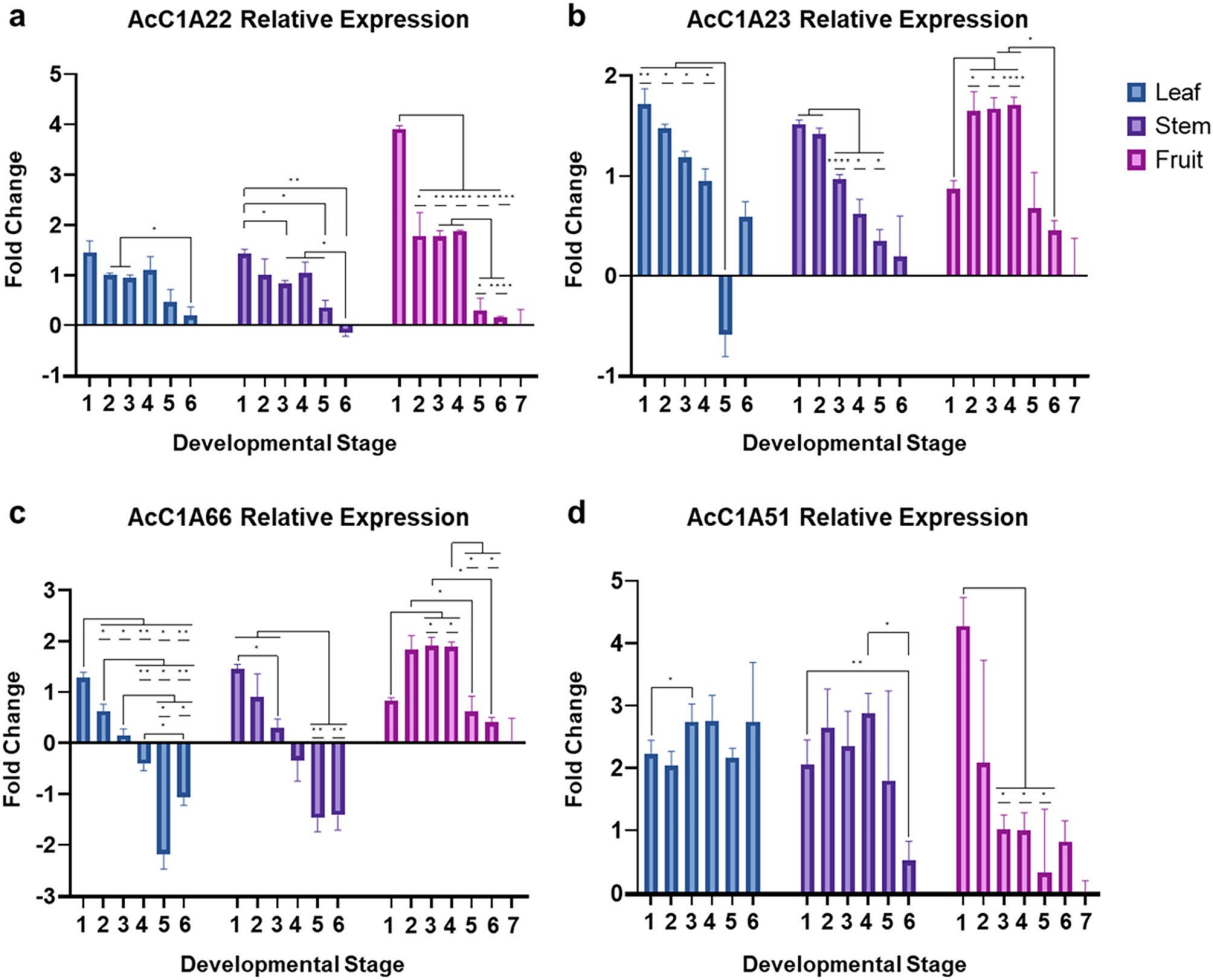
Relative expression results for RT-qPCR genes (**a**) ACMD2v2_05.00101, (**b**) ACMD2v2_05.00102, (**c**) ACMD2v2_19.05600, and (**d**) ACMD2v2_10.11600. The bars represent the SD of fold change for three biological replicates. Statistically significant differences between stages are indicated with asterisks (“*” = p-value < 0.05, “**” = p-value < 0.01, “***” = p-value < 0.001, “****” = p-value < 0.0001).

For all genes tested except *AcC1A51*, overall gene expression decreased over time, regardless of tissue type (Fig. 4a–d) and was usually higher in earlier stages of development. However, despite having similar overall expression patterns across developmental stages, *AcC1A22*, *AcC1A23*, and *AcC1A66* were all more highly expressed in fruit tissue than in leaf or stem tissues (Fig. 4a–c, Supplementary Table S11).

A two-way ANOVA test for developmental stage and tissue type confirmed that both stage and tissue type significantly impacted the expression of *AcC1A22*, *AcC1A23*, and *AcC1A66* genes, however, only stage significantly impacted the expression of *AcC1A51* (Supplementary Table S12).

For gene *AcC1A22*, expression levels generally decreased over time for all tissues. Fruit tissue displayed notably higher expression levels than leaf and stem tissues at stages 1 through 4, however, a drastic decrease in expression level was observed at stages 5 through 7 (Fig. 4a). A significant decrease in expression was observed in fruit from stage 1 to 2, fruit from stage 4 to 5, and stem from stage 5 to 6. Gene expression levels were very similar for all tissues in stages 5 and 6. For gene *AcC1A23*, expression levels started relatively high and gradually decreased during development in leaf and stem tissues (Fig. 4b), and the highest expression levels in fruit tissue occurred from stages 2 to 4. A significant increase in expression was observed in fruit from stage 1 to 2 and significant decreases were observed in stem from stage 2 to 3 and in leaf from stage 4 to 5. For gene *AcC1A66*, expression levels followed a similar pattern as gene *AcC1A23*, with a gradual decrease in expression during development in leaf and stem tissues and having the highest expression levels in fruit tissue occurring from stages 2 to 4. A significant decrease in expression was observed in leaf from stage 1 to 2 and in fruit from stage 4 to 5 (Fig. 4c). Finally, gene *AcC1A51* had somewhat consistent expression levels across stages for leaf and stem tissues. An exception is a noticeable decrease in expression levels for the last stage of stem tissue (stage 6) (Fig. 4d). Expression patterns for fruit tissue were similar to those for gene *AcC1A22*, with the highest expression level in stage 1 and decreasing over time. Although some significant differences were observed between different stages, expression levels did not significantly change from one stage to the next (e.g., from stages 1 to 2, 2 to 3, etc.) for any given tissue.

Overall, the relative expression results for genes *AcC1A22*, *AcC1A23*, and *AcC1A66* validated the expression patterns observed in the RNA-seq expression data, which indicated that *AcC1A22* and *AcC1A66* were more highly expressed in fruit than in stem and leaf tissues, while *AcC1A23* had more consistent expression levels across fruit, stem, and leaf tissues (Supplementary Table S8). Additionally, RT-qPCR expression results agree with previous findings that have found bromelain genes to be more highly expressed in ripening fruit tissues (stages 1–6) than in mature fruit tissues (stage 7)^17, 18^.

## Discussion

Using a newly published improved genome assembly and annotation for pineapple^42^, a larger number of C1A family protease-encoding genes were identified compared to recent studies on this gene family^18, 19^. A total of 71 C1A protease genes were identified in the pineapple MD2 v2 genome, compared to the 61-62 previously identified in the F153 v3 genome. The MD2 v2 C1As were characterized by orthologous and phylogenetic analysis. This study presents a systemic approach to target C1A family genes in pineapple, identify genes belonging to the bromelain subfamily, and trace their ancestry. Methods used to classify bromelain genes into subfamilies in previous studies^18^ were limited to phylogenetic clustering, which placed 44 out of 61 C1A proteases into one group (subfamily VI). In contrast, the current study utilized multiple methods for classifying C1A proteases into subgroups, including orthologous, phylogenetic, and proteomic analyses. Out of the 62 C1A protease genes previously identified in the ‘F153’ pineapple genome, 43 were orthologous to C1A proteases identified in ‘MD2’ as part of the current study. The differences in predicted number and variety of C1A proteases between this and former studies are likely explained by improvements to the pineapple genome assembly and differences in gene content between pineapple genotypes. In addition, sequenced bromelain proteins in their analyses to aid in the identification of relevant subgroups were provided. The integration of orthologous and phylogenetic analysis plus integration of previously functionally characterized bromelain genes in these analyses led to classification of the number of putative bromelains into a single orthogroup (189) and primarily one phylogenetic clade (XIII) and the ability to trace their evolutionary history. Duplication mechanism and syntenic analyses revealed what are likely the most ancestral members of orthogroup 189, the orthogroup associated with bromelain subfamily enzymes. We were able to trace the ancestry of 3 genes (*AcC1A61*, *AcC1A62*, *AcC1A63*) within orthogroup 189. To note, none of the 9 species included in the orthologous analysis had > 1 representative gene in group 189, which makes us hypothesize that these three genes are older members of orthogroup 189 and an expansion in orthogroup 189 occurred within the pineapple lineage. A loss of representative genes within this orthogroup across all non-pineapple species would be less likely than a single-species expansion. Genes in orthogroup 189 have largely undergone positive selection, leading to diversification of this orthogroup. This diversification of orthogroup 189 possibly led to the acquisition of new functions and emergence of bromelain subfamily genes in pineapple.

The results presented here also provide valuable information regarding what bromelain proteins are present in commercially available products (AcC1A22, AcC1A23, AcC1A28, AcC1A31) and the gene sequences encoding them. Interestingly, one of the likely bromelains identified in the MD2 v2 predicted protein sequences, AcC1A23, was very close to stem bromelain on the phylogenetic tree and was the most abundant C1A protease protein identified in both samples by proteomic analysis. The second most abundant likely bromelain protein was AcC1A31, which was also in clade XIII on the phylogenetic tree (Fig. 2). The least abundant likely bromelain proteins were AcC1A22 and AcC1A28, which were also in clade XIII. The phylogenetic position and relative abundances of the four likely bromelains out of seventy-one total C1A family proteases identified in this work strongly suggests that (1) the *AcC1A22, AcC1A23, AcC1A28,* and *AcC1A31* genes represent bromelain enzymes encoded in the MD2 v2 genome and (2) these proteins are likely responsible for at least some of the proteolytic activity reported for commercial bromelain products. Further work will need to be done to determine the proteolytic activity of individual bromelain enzymes.

RNA-seq analysis indicated that the identified bromelain genes (*AcC1A22, AcC1A23, AcC1A28, AcC1A31*) did not show tissue-specific expression, but were highly expressed and up-regulated in fruit and stem tissues as well as being present in commercial bromelain samples as shown in the proteomic analysis. Similarly to previous findings^17, 18^, these genes were also more highly expressed during fruit ripening than in mature fruits and were hypothesized to play a role in fruit ripening based on their expression profiles. Despite being named stem and fruit bromelain, the bromelain genes identified in this study are not specifically expressed in only these tissues but were expressed in all tissues (Supplementary Table S8). The gene expression results indicate that the commonly used labels “fruit” and “stem” bromelain do not necessarily reflect the phylogenetic origin of these enzymes or tissue specific expression, but are likely named according to the tissue they are extracted from for commercial use. For example, as highlighted by the RNA-seq and RT-qPCR results, gene *AcC1A23*, most likely stem bromelain, was not differentially expressed between fruit and stem tissues, but was up-regulated in these tissues compared to the other tissues. Further work will need to be done to determine if a different subset of putative bromelain proteins or if different protein abundances exist in purified fruit extracts.

Given the duplication modes and gene expression levels identified among C1A family gene pairs here, as well as the biological functions of C1A family proteins in preventing herbivory and disease^12^, external factors were likely to be the main drivers of positive selection for this gene family in pineapple. Expansion of the C1A protease family in pineapple may have contributed to a higher degree of herbivory and disease resistance. A greater number of proteolytic enzymes would benefit plant species accessible to both ground- and tree-dwelling herbivores, and prevent the singular fruit from being consumed by fungi or bacteria. Indeed, in previous research bromelain has been proven to reduce fungal and bacterial diseases^15, 39^, and many people find that eating large amounts of fresh pineapple fruit results in stinging, uncomfortable oral sensations that can be attributed to bromelain activity^40^.

The results of this study could be utilized for a variety of applied science, including transgenic approaches to developing plant bioreactors for high bromelain production, nutritional studies that more precisely characterize bromelain function as a bioactive molecule, and targeted therapeutic studies.

## Methods

### Identification and manual curation of C1A family genes

The predicted gene and protein sequences from the recently published *A. comosus* MD2 v2 genome were used for identifying genes encoding C1A proteases in pineapple^42^. First, protein domains were obtained for the predicted protein sequences by querying them against the InterPro database^43^ using the InterProScan feature of Omicsbox v.2.1.10^44, 45^ with default parameters. Then, C1A family proteases (EC: 3.4.22) were identified among the annotated protein sequences by searching for the C1 peptidase (IPR000668) and/or inhibitor I29 (IPR013201) domains.

C1A proteases lacking either the C1 peptidase domain or inhibitor I29 domain were further examined to ensure correct structure of the corresponding gene. As the first step for this analysis, Illumina reads from the NCBI SRA database for Bioprojects 483249^41^ and 552841^18^ and PacBio Iso-seq reads for MD2 v2^42^ were aligned against the MD2 v2 genome. Illumina reads were aligned using STAR and setting the following parameters: --outSAMstrandField intronMotif --outSAMattrIHstart 0 --outFilterMismatchNmax 2 --outSAMtype BAM SortedByCoordinate^46^. Iso-seq reads were aligned using GMAP aligner with the following parameters: --min-identity = 0.99 --min-trimmed-coverage = 0.95 --nosplicing^47^. Genome, CDS, and alignment tracks were loaded in IGV^48^ to inspect presence of discrepancies between the structure of the predicted genes and the read alignments. Genes with notable reads aligned outside of the predicted CDS region were selected for re-prediction of gene structure.

To re-predict these genes, the genomic sequence spanning the gene prediction, plus 1kb of flanking sequence on either side were extracted and used as input for eukaryotic gene finding in Omicsbox^49^. Re-predicted genes were scanned for the presence of C1 peptidase and/or inhibitor I29 conserved domains as described above. In those cases where the structure of the re-predicted gene gained a C1 peptidase or inhibitor I29 domain that was missing in the original prediction, the re-predicted genes were accepted as final gene structure. Additionally, to search for potential isoforms, all identified C1A genes were manually inspected in IGV following the same method used for detecting mis-predicted C1A proteases. Finally, SignalP v.6.0^50^ was used to identify signal peptides in all C1A protein sequences.

### Phylogenetic and evolutionary analysis

Phylogenetic and orthologous relationships and mode of duplication of the predicted protease C1A genes were studied to elucidate their origin and to associate specific clusters to sequenced bromelain proteins and functionally annotated bromelain genes. For this analysis ortholog clustering of C1A genes was performed using OrthoMCL v.2.0.9 (https://orthomcl.org) with predicted genes from three pineapple genomes (MD2 v2, MD2 v1, and F153 v3), multiple other species of varying degrees of relatedness (*Arabidopsis thaliana, Carica papaya, Musa acuminata, Oryza sativa, Sorghum bicolor, Solanum lycopersicum*, *Vitis vinifera*, and *Zea mays*)^42, 51–59^, as well as known bromelain sequences (proteins and CDS) obtained from Uniprot (O23791, O24641, P14518, Q7DNA3, and P80884; one published and four direct submission)^28^, and an additional published protein sequence for ananain^26^.

For phylogenetic analysis, nucleotide sequences of the MD2 v2 C1A proteases and known bromelain sequences (see previous section) as well as orthologous proteins from *Arabidopsis thaliana* and *Oryza sativa* were aligned with ClustalW (https://www.clustal.org/) using default parameters. The published protein sequence for papain was also included as the outgroup in this analysis. Subsequently, the phylogenetic tree was constructed using MEGA-X v.10.2.6 (http://www.megasoftware.net) via the Maximum-likelihood (ML) method and Tamura-Nei model. Node robustness was estimated using the bootstrap method with 100 replications. Clade numbers were assigned to groups in the phylogenetic tree. The C1A proteases from the MD2 v2 genome annotation that were in the same orthogroup and phylogenetic clade as known bromelain sequences were considered to be putative bromelains.

Mode of duplication of predicted C1A genes in the pineapple MD2 v2 genome was assessed using DupGen_finder^37^ (default parameters) with the *O.sativa* Nipponbare reference genome^54^ as the outgroup species. The DupGen_finder-unique perl script was then run to assign all duplicate genes to a unique duplcation mode. Duplicated gene pairs were identified and classified into one of five categories: whole genome or segmental duplication (WGD), tandem duplication (TD), proximal duplication (PD), and transposed duplication (TRD) pairs. The nonsynonymous to synonymous mutation (*Ka/Ks*) ratio for duplicate gene pairs was calculated using the simple *Ka/Ks* calculator tool in TBtools v.1.09876 software^60^. The ancestry and syntenic relationships between C1A genes in pineapple, rice, and *Arabidopsis* were analyzed using the python version of the Multiple Collinearity Scan tool (MCScan)(--cscore=.80 -n 2)^61^.

### RNA-seq analysis

Available RNA-seq data were used to study the patterns of expression among identified C1A genes. Pineapple RNA-seq data available in the NCBI SRA database for Bioprojects 483249 and 552841 were downloaded and used for differential expression analysis. NCBI Bioproject 483249 is comprised of Illumina transcriptome data representing 11 different pineapple tissues (bract, core, flower disk, leaf, ovary wall, ovule, placenta, receptacle, root, sepal, stem) collected from cultivar ‘Shen Wan’ at multiple stages during fruit development. NCBI Bioproject 552841 is comprised of Illumina transcriptome data representing 16 different pineapple tissues (flower, fruit at multiple stages, leaf, androecium, gynoecium) collected from two pineapple species (*A. comosus* and *A. bracteatus*).

Expression level of the C1A genes, measured as fragments per kilobase of exon per million reads mapped (FPKM), and their differential expression between tissues were evaluated. Initial alignment of Illumina reads to MD2 v2 (phase 0) predicted CDS sequences and raw count data were obtained using RSEM v.1.3.3 and the --bowtie2 parameter^62^. DESeq2 v.3.15^63^ was used for pairwise differential expression analysis. A C1A gene was considered to be not expressed if it had FPKM < 2 and C1As with FPKM ≥ 2 were considered to be expressed. NCBI Bioprojects 483249 and 552841 were each used independently for differential expression analysis (up or down regulated genes).

### Total RNA isolation and RT-qPCR

RT-qPCR was performed to further investigate the pattern of gene expression of selected putative bromelain genes across different tissues and developmental stages. Based on results of phylogenetic and gene expression analysis, four C1A protease genes (*AcC1A22, AcC1A23, AcC1A66*, and *AcC1A51*), including three putative bromelain genes identified by orthologous and phylogenetic clustering, were selected for RT-qPCR. A commercial fresh-fruit market variety, MD2, was used for this experiment. Pineapple MD2 plants were grown at the Dole plantation field in La Ceiba, Honduras.

For RNA extraction, fresh tissues of field grown MD2 pineapples were harvested and placed immediately on dry ice. Leaf, stem, and fruit tissues were harvested at seven different developmental stages starting from early fruit development to fully matured fruit as follows: 1 = cone (young fruit before flowering), 2 = first flower (lower 1/3 of inflorescence flowering), 3 = mid flower (middle 1/3 of inflorescence flowering), 4 = late flower (upper 1/3 of inflorescence flowering), 5 = green (green shell color), 6 = normal harvest (fruit harvested according to production schedule), and 7 = full maturity (ripe and ready for consumption). These tissues correspond to BBCH codes 507, 601, 605, 607, 705, 709, and 809^64^ for stages 1–7, respectively. Three biological replicates were collected for each sample. Note that for stage 7 only fruit tissue was collected.

Total RNA was extracted from collected tissues using the Qiagen RNeasy plant mini kit (Qiagen, Germany). RNA integrity was evaluated on a 1.0% agarose gel, quantified using Qubit 2.0 fluorometer (Invitrogen, USA), and purity was tested using NanoDrop (Thermo Fisher Scientific, USA).

RNA (~ 600 ng) was synthesized into cDNA by using the SuperScript III First-Strand Synthesis System (Invitrogen, USA) through a one-step method. RT-qPCR was performed on a LightCycler 480 II (Roche, Switzerland) using SYBR Green qPCR Master Mix (Thermo Fisher Scientific, USA) with three biological and technical replicates for each gene. Expression levels of the analyzed C1A genes were normalized to the transcript levels of pineapple β-Actin. Published primer sequences that have high specificity for the pineapple β-Actin gene were used in this experiment^65^. The relative expression levels of genes were calculated using the 2^(− ΔΔCt) method with full maturity (stage 7) fruit as the reference sample. The GraphPad Prism 9 (Dotmatics, USA) software was used for conducting statistical analyses (significance P < 0.05) (http://www.graphpad.com/).

### C1A protein content analysis of pineapple stem

Proteomic analysis was performed to determine which of the MD2 v2 C1A proteases were present in commercial bromelain products derived from pineapple stem (Fondazione Toscana Life Sciences, Italy).

Two different commercially available bromelain extracts from pineapple stem (B1, Sigma-Aldrich, USA, Cat. #B5144 and B2, Galeno Srl, Italy, Cat. # 5280) were weighed and dissolved in warm water for 15 min at a final concentration of 1 mg/ml. 80 μg of bromelain extracts were reduced with 100 mM dithiothreitol (DTT) at 60 °C for 30 min and alkylated in the dark with 100 mM of iodoacetamide (IAA) at room temperature for 30 min. Next, each sample was processed by adding trypsin (Promega, USA) using an enzyme-to-protein ratio of 1:40 and incubated at 37 °C overnight. Following digestion, all reaction mixtures were acidified with 1% FA in order to inhibit any remaining enzyme activity.

Digested samples were desalted using OASIS cartridges (Waters, USA), brought to dryness, and reconstituted in 0.1% formic acid in water to a final concentration of 1 μg/μl. LC-MS/MS analyses were performed using a Q-Exactive HF-X Orbitrap mass spectrometer (Thermo Fisher Scientific, USA).

The peptide separation was carried out at 35 °C column oven temperature using a PepMap RSLC C18 column, 75 μm × 15 cm, 2 μm, 100 Å (Thermo Fisher Scientific, USA) at a flow rate of 300 nl/min. Mobile phases of 0.1% formic acid in water (A) and 0.1% formic acid in acetonitrile (B) were used to separate peptides, using 5% B and 95% A, changing to 90% B and 10% A at 120 min. These experiments were performed using a data dependent acquisition (DDA) setting to select the “top twelve” most-abundant ions for MS/MS analysis. Protein identification was performed with Proteome Discoverer 2.5 (Thermo Fisher Scientific, USA) and Sequest algorithm (default parameters) using the entire set of MD2 v2 predicted protein sequences as a custom database for the peptide sequence queries and the following settings: precursor mass tolerance of 10 ppm, fragment mass tolerance of 0.2 Da, and trypsin specified as the digesting enzyme with 2 missed cleavages allowed. The results were filtered for high confidence peptides with FDR < 5% and matching with ≥ 3 peptide sequence tags. Putative bromelain proteins (see “Phylogenetic and evolutionary analysis” section) that were present in either of the commercial bromelain products analyzed were considered to be likely bromelains.

## Supplementary Information

Supplementary Figures.

Supplementary Tables.

## Data Availability

The datasets analyzed during the current study are available in the NCBI repository (https://www.ncbi.nlm.nih.gov/) under the BioProject IDs PRJNA719415 (https://www.ncbi.nlm.nih.gov/bioproject/?term=PRJNA719415), PRJNA10719 (https://www.ncbi.nlm.nih.gov/bioproject/10719), PRJNA122 (https://www.ncbi.nlm.nih.gov/bioproject/PRJNA122), PRJNA483249 (https://www.ncbi.nlm.nih.gov/bioproject/?term=483249), and PRJEB33121 (https://www.ncbi.nlm.nih.gov/bioproject/?term=552841). See “Methods” section for details.
