# Supplementary material for: Identification of bromelain subfamily proteases encoded in the pineapple genome
Source: Sci Rep. 2023 Jul 18;13:11605. doi: 10.1038/s41598-023-38907-y (PMC10354200; doi:10.1038/s41598-023-38907-y)
Supplement: Supplementary file 1 — Supplementary Figures. [file 41598_2023_38907_MOESM1_ESM.docx]

**
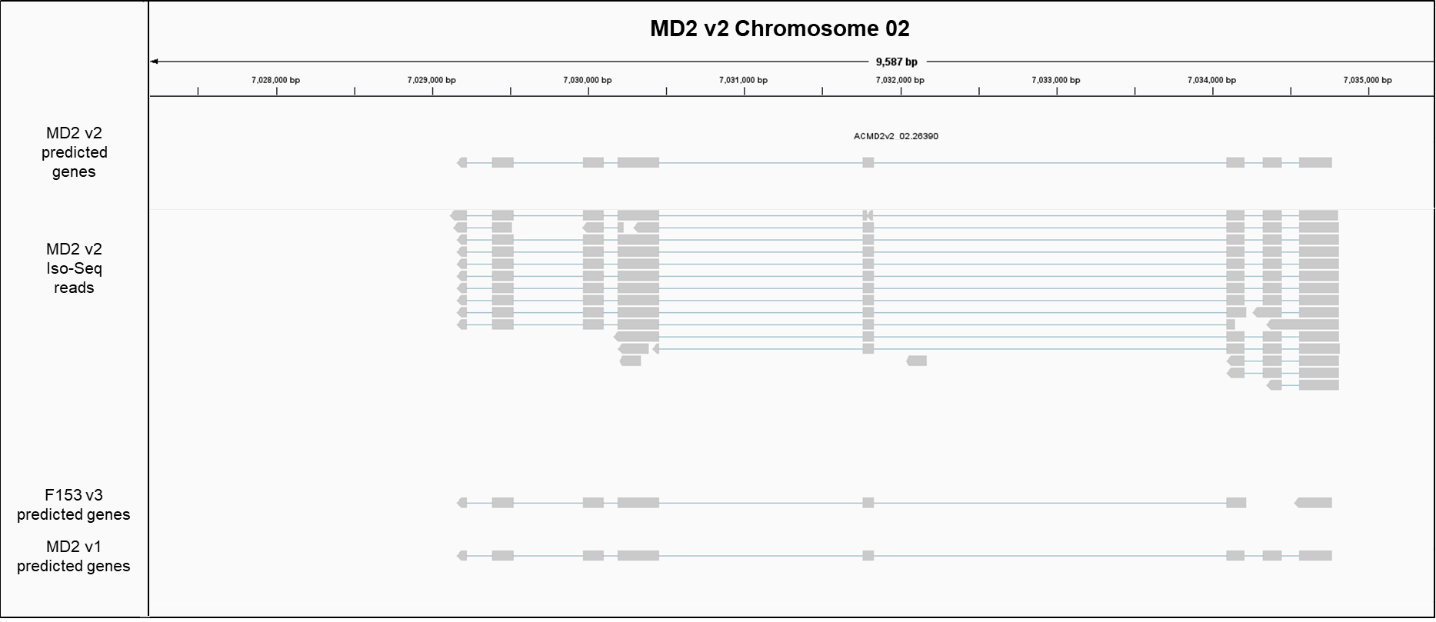
**

**Supplementary Figure 1.** Predicted structure of a C1A family protease gene in MD2 v2. Alignment of MD2 v2 predicted genes, PacBio Iso-Seq reads derived from MD2, and predicted genes from previous pineapple genomes (F153 v3 and MD2 v1) to the MD2 v2 genome validates the correctness of predicted gene structures of C1As in MD2 v2. In this example, one gene predicted in MD2 v2 was predicted as two different genes in F153 v3. IsoSeq data supported the presence of a single gene model for this locus.


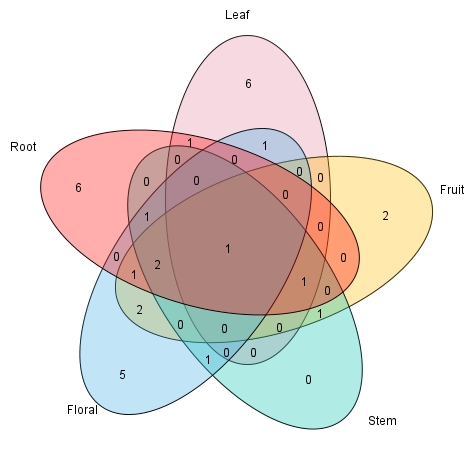


**Supplementary Figure 2.** Venn diagram depicting expression patterns of tissue-specific papain family genes identified in this study. A total of 31 papain family genes had tissue-specific expression when evaluated by FPKM. “Floral” category included multiple distinct tissues that make up the entire flower (e.g., flower disk, bracts, sepals, ovary wall, ovules, placenta, petals, stamen, style, and receptacle), therefore genes in this category may not be expressed in all individual floral tissues.
